# University of Pennsylvania

**Published:** 1847-10

**Authors:** 


22. *University of Pennsylvania.*—James B. Rogers, M.D., we are happy to announce, has been appointed professor of Chemistry in the Medical Department of this University. Prof. Rogers is an eloquent lecturer and accomplished chemist, and will well sustain the reputation of the school. —*Med. News and Library.*

